# Metabolic and appetitive regulation of adipocyte mass during treatment of obesity

**DOI:** 10.1111/joim.70045

**Published:** 2025-11-21

**Authors:** Jonathan Q. Purnell, Carel W. Le Roux

**Affiliations:** ^1^ Department of Medicine Oregon Health & Science University Portland Oregon USA; ^2^ Diabetes Complications Research Centre University College Dublin Dublin UK; ^3^ Diabetes Research Centre Ulster University Belfast UK

**Keywords:** hypothalamus, obesity, regulation of adipocyte mass, set point, weight maintenance phase, weight reduction phase

## Abstract

Adipose mass is homeostatically maintained within a narrow range despite fluctuations in daily calorie intake and activity levels. Constituting an adipose mass “set point,” this homeostatic regulation includes sensing mechanisms in the form of hormones reflecting caloric intake that serve as mediators of appetitive behaviors and adipose mass amount; integrating centers in the brain and brainstem; and response or effector systems. During adipose mass fluctuations beyond daily short‐term changes, typically during low‐calorie dieting, these effector systems include adaptive responses in metabolism (energetics), hormone production, and appetitive behaviors that resist further loss and restore adipose mass to baseline. Although our understanding of the disease of obesity is still evolving, within the context of this paper we consider the disease a manifestation of the pathophysiological processes when the expression of leptin resistance leads to the establishment of a new, higher adipose mass set point. Effective obesity therapies lower the adipose mass set point by improving appetite control and preventing the normal adaptive responses that lead to weight regain, effectively establishing a new adipose mass set point at a lower, healthier level. Conveying this biology to patients with obesity provides them with an understanding of their disease state, why drug and surgical treatments in combination with lifestyle are necessary for most people, and the mediators of the changes in appetitive behaviors expected from effective obesity therapies. Future research will need to advance the evidence base that supports this theoretical framework and generate even deeper insights into the disease of obesity.

## Graphical abstract

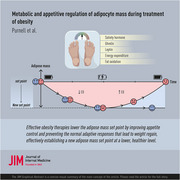

## Introduction

**Fig. 1:**
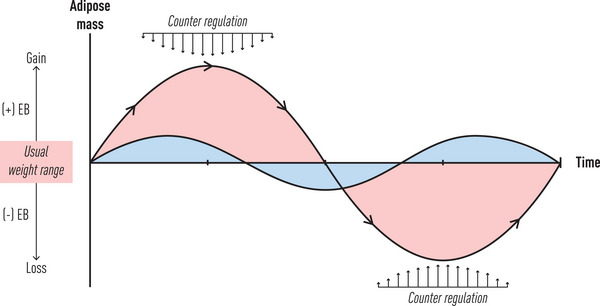
Short‐ and long‐term adipose mass regulation. Adipose mass is homeostatically regulated to maintain levels within a narrow range (usual weight range). Major deviations involving positive (+EB) and negative (−EB) energy balances are met by counter‐regulatory adaptive responses involving hormonal, appetitive, and energy metabolism systems. These responses remain in place until adipose mass is restored back to usual range, which can be within what is considered healthy or normal range, underweight, overweight, or obesity ranges.

Physiological pathways regulating adipose mass are like those that govern other vital life processes, such as core body temperature, blood glucose levels, and blood pressure. All involve some capacity to “sense” one's internal and external environments and homeostatically [1] respond to maintain those processes within a narrow range that ensures normal functioning and health. Although homeostatic responses for maintenance of euglycemia and normal blood pressure are well accepted within the scientific and medical communities and, indeed, form the physiologic basis of our current pharmacologic management of diabetes (e.g., insulin, metformin, and sodium–glucose cotransporter 2 inhibitors) and hypertension (e.g., diuretics, beta‐blockers, and angiotensin convertase enzyme inhibitors), such understanding is less intuitive for adipose mass regulation, especially in the care of patients with the disease of obesity (Fig. 1).

This review will cover the metabolic, hormonal, and appetitive adaptations when adipose mass is reduced and how they influence both patient experiences and therapeutic efficacy. Interventions used to manage patients with obesity will be compared, including lifestyle approaches, obesity medications, and metabolic–bariatric surgeries. Understanding adaptation responses to adipose mass changes will aid patient understanding of their disease, provide a framework to incorporate mechanisms of current and future obesity treatments, and will help establish the need for personalized treatment approaches.

### Review of adipose mass regulation in health and the disease of obesity

#### Adipose mass regulation: brain–gut–adipose tissue axis

At its most basic level, normal weight regulation involves communication between three principal organ systems: the central nervous system and brainstem, gastrointestinal system, and adipose tissue. As previously described, afferent hormonal signals originating from the intestinal enteroendocrine cells during nutrient digestion and absorption convey information to hypothalamic (and brainstem) centers indicating fed and fasting states as well as calories consumed during a meal [2]. The brain integrates these signals with that of leptin, levels of which indicate adipose mass (fat cell number and size) [3]. Because weight changes in adulthood predominantly reflect changes in adipose mass, leptin levels (and leptin receptor activation state) effectively denote current body weight. It should be acknowledged that central nervous system insulin levels (and receptor signaling) have been shown to act similarly to leptin as an “adiposity” signal [4]. Indeed, it is likely that other peripheral signals exist reflecting adipose, muscle, and bone mass—essentially body composition—with their own central nervous system pathways and responses. However, inclusion of these other signals is beyond the scope of this review. Instead, focus will be on the evidence for leptin's canonical role in the homeostatic regulation of adipose mass.

#### Adipose mass regulation: set point defense

Should changes in these peripheral hormonal signals indicate declining calorie intake [5, 6], adipose tissue mass [7], or both, then efferent response systems are activated by central nervous system centers to homeostatically oppose these changes and restore adipose mass to baseline (Fig. 1). These response systems constitute adaptation pathways that maintain weight despite sometimes wide fluctuations in food availability and activity levels. Collectively, the afferent signals mentioned above—integrating brain centers—and efferent pathways mediating these adaptation responses are referred to as the body weight “set point.” Although this term is often used in reference to body weight, in concept it is no different than from physiological “set points” for glucose levels, blood pressure, lipid levels, or blood oxygen content. Such set points do not represent a single “normal” value but embody a narrow range in which healthy functioning is maintained, and risk for future complications is minimal. Less appreciated but seemingly self‐evident, when adipose mass levels are in steady state, these homeostatic systems also protect against excessive increases (Fig. 1), as evidenced in numerous “overfeeding” studies showing adaptation responses opposite to those prompted during caloric restriction [8]. As discussed later, this contrasts with unwanted weight gain that a person experiences when the expression of the disease of obesity results in a rising adipose mass set point. In this situation, normal counter‐regulatory responses are not appropriately activated, which would normally limit and reverse this weight gain, instead leading to a period of positive energy balance until a new, higher adipose mass set point is established, at which point normal counter‐regulatory responses are restored [2].

#### Expression of excess adiposity: leptin resistance raises the set point

Although an oversimplification, expression of unwanted weight gain as a consequence of the disease of obesity can most easily be understood as resulting from emergence of leptin resistance [2], analogous to insulin resistance leading to hyperglycemia and type 2 diabetes. For example, to maintain euglycemia, islet cells respond to mild glucose elevations accompanying insulin resistance with increased insulin secretion and cellular hypertrophy, establishing a higher blood insulin level (hyperinsulinemia) [9]. If insulin resistance is not corrected and islet cells eventually become unable to adequately compensate, also known as “relative insulin deficiency,” then patients progress through stages of prediabetes and diabetes. Islet cell hypertrophy and hyperinsulinemia are the result, not cause, of type 2 diabetes. With leptin resistance, a relative leptin‐deficient state is sensed by the brain (most often by signaling defects downstream from arcuate neurons that contain leptin receptors) [9], leading to an accumulation of fat tissue and establishment of a higher leptin level whereby normal central leptin signaling is again established, albeit at a new, higher adipose mass [2]. Progressing through stages of adipose mass gain requires failure of the adipose mass set point to adequately compensate by suppressing appetite and increasing energy expenditure. In other words, developing obesity occurs because the central nervous centers permit it, not because of neglect or a lack of willpower on the part of the affected individual. In this regard, unwanted adipose mass gain is both a manifestation of the disease of obesity and—by mediating detrimental immune, metabolic, and mechanical effects—its proximate cause.

#### Excess adiposity: defending a higher set point and need for lifelong therapies

Once a higher adipose mass and leptin level set point is established, the central brain centers governing body weight will sense and effectuate adaptive responses to afferent signal changes reflecting declines in calorie intake and adiposity levels at the new higher set point, just as they did at the lower one. The directionality of this rise in adiposity set point in people with obesity is most frequently one way, similar to those who progress from normal glucose and blood pressure physiologies to developing type 2 diabetes and hypertension. To date, no amount of weight loss duration through lifestyle (dieting and exercise) interventions alone has been shown to “reset” the homeostatically defended adipose mass to a lower level [10]. The inevitable adipose mass regain, often referred to as weight “yo‐yoing,” does not reflect a relapse of the obese state, which would imply a remission or cure in the first place but is instead indicative of the chronic (sometimes progressive) nature of the disease of obesity necessitating lifelong therapies.

The adaptation responses that lead to weight maintenance of an adipose mass set point involve the same systems regardless of whether a person is in a healthy or unhealthy weight range and can be broadly categorized as metabolic (energetic), hormonal, and appetitive (behavioral). In the following sections, evidence of these adaptation responses will be discussed, specifically in reference to the obese state unless otherwise indicated.

### Metabolic (energetic) adaptations

Daily energy expenditure comprises energy necessary to maintain all cellular processes (basal metabolic rate when asleep, resting energy expenditure when awake), the energy needed to digest food (thermic effect of food), adaptive thermogenesis (shivering), and activity‐associated thermogenesis [11]. The most flexible of these energy expenditure components is activity, which can be categorized broadly as exercise‐associated activity and non‐exercise‐associated thermogenesis [12, 13].

**Fig. 2:**
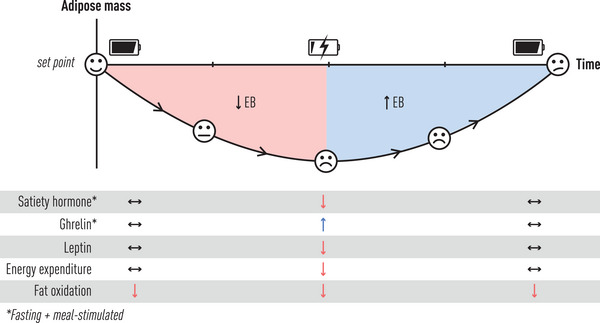
Metabolic, hormonal, and appetitive adaptive responses to low‐calorie‐induced weight loss. Facial expressions indicate patient experiences from baseline adipose mass set point to determined during active negative energy balance (EB), to worried/frustrated during positive energy balance induced weight regain, to puzzled once baseline adipose mass is restored. See text for full descriptions.

For patients with the disease of obesity, if adipose mass reduction and maintenance were as simple as “moving more,” then treatment would be very straightforward. However, daily energy expenditure undergoes adaptation in response to changes in activity demands as well as during low‐calorie diet‐induced weight loss. Regarding daily energy expenditure changes in weight‐stable individuals, evidence for adaptation is strongest for activity: exercise‐associated activity and non‐exercise‐associated thermogenesis. As exercise‐associated activity levels increase, a threshold is reached in which further increases result in proportional decreases in non‐exercise‐associated thermogenesis activities [13], keeping daily energy expenditure and adipose mass stable. For those with obesity participating in a low‐calorie diet‐induced weight loss program, studies dating back 40 years have shown an approximately 15%–20% reduction in daily energy expenditure that is in addition to the reduction expected from lower lean and fat mass energy needs (Fig. 2) [14, 15, 16, 17]. Accompanying and possibly contributing to this increase in energy efficiency are reports of altered metabolic partitioning of fuels in which the oxidation of free fatty acids liberated by fat cells is impaired in those with obesity and fails to improve with weight loss (Fig. 2) [18]. The practical implication of these findings is that following weight loss, 15%–20% of daily calorie intake calculated to meet the patient's needs at their new, lower weight would, in actuality, go back into fat storage. Such individuals would need to further reduce calorie intake, varying in amount by patient, to maintain their lost weight. Long‐term follow‐up of such low‐calorie diet‐induced weight loss studies shows that this energetic (metabolic) adaptation response remains intact and does not “adjust” to the new lower adipose mass over time [17, 19]. In fact, only upon restoration of the previous adipose mass (pre‐weight loss weight) does the metabolic adaptation response resolve.

### Hormonal adaptations

Hormonal adaptations to low‐calorie diet‐induced weight loss fall into several categories, including hypothalamic–pituitary changes, changes in the sympathetic nervous system, and gut hormones (Fig. 2).

#### Hypothalamic–pituitary axes

The best studied hypothalamic–pituitary hormone changes to low‐calorie diet‐induced weight loss involve reductions in the hypothalamic–pituitary–thyroid (HPT) axis. Following 10% and 20% total body weight loss, blood levels of T3 and T4 decline without a compensatory rise in TSH, indicating a secondary (hypothalamic) hypothyroid state [20]. Preclinical studies and phenotypic descriptions of humans with leptin receptor deficiency demonstrate central leptin control of HPT signaling [21, 22]. Indeed, leptin replacement after weight loss can restore thyroid hormone levels toward normal [20]. Impairment of the HPT axis with weight loss could theoretically contribute to the metabolic adaptation described above to slow down weight loss and promote weight regain (and contribute to feeling cold and hair loss). However, studies of thyroid hormone replacement following a low‐calorie diet‐induced weight loss have shown only partial restoration of this adaptation [23] without demonstrable long‐term impact on weight loss compared to placebo [24], and the possible acceleration of muscle protein loss when administered in supraphysiological doses [25].

Another hypothalamic–pituitary axis impacted by obesity and weight loss involves the reproductive system. Women with obesity can develop oligomenorrhea, often diagnosed as polycystic ovarian disease, thought to be mediated by central suppression of gonadotrophs (hypothalamic hypogonadism) due to increased aromatase activity by enlarged adipose tissue [26], increased ovarian production of androgenic steroids under the influence of increased insulin levels in subsets of women [27], and/or augmented LH production by elevated leptin levels [28], alone or in combination. This reproductive cycle disruption is likely a consequence rather than a cause of obesity as adipose mass reduction helps restore normal periods and fertility [29]. On the other hand, excess adipose tissue loss that induces low or effectively deficient leptin levels can lead to hypogonadotropic hypogonadism (amenorrhea) that is partially reversed with leptin replacement [30]. To date, no evidence supports the involvement of either the hypothalamic–pituitary adrenal (cortisol) or growth hormone axes in the adaptive response to weight loss.

#### Sympathetic nervous system

Preclinical studies have linked leptin and melanocortin signaling with the sympathetic nervous system. Enhanced leptin signaling and melanocortin signaling increase sympathetic nervous system outflow, which can increase heart rate, blood pressure, and energy expenditure, while impairing insulin secretion in rodents [31, 32, 33]. Accordingly, low‐calorie diet‐induced weight loss and resulting reduction in leptin levels have been shown to reduce markers of sympathetic nervous system activity and heart rate, both of which can be reversed with leptin replacement [34]. These collective downregulations of hypothalamic and sympathetic systems in response to low‐calorie induced weight loss, and their limited reversal with leptin replacement simultaneously with partial restoration of reduced energy expenditure, indicate their potential roles in mediating the adaptive reduction in energy metabolism when adipose mass is reduced and leptin levels decline. Unfortunately, these leptin‐induced reversals in adaptive hormone and metabolic responses are not sufficient for leptin to be used as a monotherapeutic obesity medication [35].

#### Gut hormones

Gut hormone responses during nutrient absorption play several pivotal roles in weight regulation and the adaptation response to low‐calorie‐induced weight loss. Meal stimulation of intestinal enteroendocrine cells releases a pattern of hormones and suppresses ghrelin production from the stomach and duodenum, followed by the restoration of levels to baseline before the next meal [36, 37, 38]. These meal‐to‐meal changes in hormone levels signal to the brain via both blood and enteric vagal nerve receptors, the fed and fasting states, the number of meal calories contained in that meal [5, 37] and serve as the biologic basis for appetitive behaviors (see below) [39, 40, 41]. The effect of these hormonal responses on appetitive behavior should be viewed within the context of whether the adipose mass regulation is in homeostasis (resulting in stable appetitive behavior), below the set point (increased appetitive behavior), or above the set point (reduced appetitive behavior) (Fig. 1).

Most of the enteric hormones released during meal consumption convey a sensation of fullness and are referred to as satiety factors. Levels of the orexigenic hormone ghrelin are initially suppressed with meal initiation, reaching a nadir approximately 90 min later before rising back to baseline [42]. These divergent responses are consistent with a regulatory model in which the balance of appetitive signals during a meal favors limiting total calorie intake. Whether return of satiety hormone levels to baseline, the subsequent rise in post‐meal ghrelin levels, or both are necessary to initiate the next meal is not known [37]. Because the amount of satiety hormone released and the degree of ghrelin suppression are proportional to the number of calories in the meal [5, 37], the integration of these signals is likely the means by which the subcortical areas of the brain centers “sense” meal‐related calorie content. Once the brain determines that the requisite calorie intake is reached, then the satiety signal is converted to one of satisfaction (satiation) with a behavioral response of voluntary meal cessation [43]. During ad libitum intake, satiation typically occurs at roughly 60% of maximal fullness on a scale of 1–10 [44, 45, 46].

Subsequently, voluntary calorie restriction (e.g., fasting, low‐calorie and very‐low‐calorie diets, and/or portion control) in which attained satiety hormone levels [47] and initial ghrelin suppression are reduced, followed by a sooner return to baseline values [37], would be predicted to lead to a patient experience characterized by remaining unsatisfied at the predetermined meal amount, still feeling hungry, and/or becoming hungry again more quickly after the meal. Indeed, studies have shown that following a period of low‐calorie diet‐induced weight loss, patients with obesity generate lower levels of satiety hormones [48] and ghrelin levels [48, 49] increase in response to the same meal‐calorie intake (Fig. 2). The reason for this is unknown, but it is currently thought to represent an important adaptation to the weight loss that tips the hormonal balance in favor of increasing meal‐related food intake and meal/snack frequency. Leptin levels play an important modifying role in this appetite hormone signaling. Animal models show synergism between leptin and satiety hormones signaling on appetitive behaviors. Compared to subtherapeutic administration of either alone, the combination of a satiety hormone and leptin together further lowers food intake [50, 51]. For patients going through a low‐calorie diet program in whom satiety hormone levels are reduced after weight loss as described above, without a change in leptin resistance, lower leptin levels accompanying adipose tissue mass reduction serve to further weaken the meal‐related fullness signals and strengthen the ghrelin hunger signal (Fig. 2). The net effect of this for animals and humans alike, whether lean or with obesity, is to increase meal size, meal frequency, or both as part of the adaptation in changes in gut hormone and leptin levels and receptor signaling following diet‐induced weight loss.

### Appetitive (behavioral) adaptations

#### Appetitive behaviors are driven by endogenous biological signals

An important insight from the scientific discoveries described above of nutrient‐stimulated gut hormone release and suppression is that appetitive behaviors governing meal size and frequency are driven by these endogenous biological signals. When a meal is initiated, when it is terminated, the act of seeking food, or tolerating (or not) prolonged periods of fasting are governed by the balance of appearance or disappearance of these hormonal signals and the activation state of their central receptors. What this practically means for a patient is that although it is commonly thought that when and how much food we eat is under our conscious control, in reality our “behaviors” are directed by these endogenous signals. Stated differently, food consumption is not in and of itself a behavior, but a consequence of behaviors driven by biology, not volition. These biological signals cannot be consciously controlled and will persist until action is taken. In this context, hunger is analogous to activation of thirst centers in response to plasma volume loss [43]. Thirst and water seeking are also behavioral responses to dehydration that restore plasma volume through increased fluid intake. However, thirst cannot be willfully ignored. Thirst and the motivation to seek fluids will remain until water is found, and the amount of water needed to restore normal hydration is not under conscious control either. A person cannot predetermine that they will be satiated with just 20 mL of water. If more is needed, then they will continue to be thirsty and seek water until the body registers enough consumed to restore plasma volume homeostasis, at which point the thirst drive abates.

What has become apparent is that many gut hormones and other intestinal factors known to serve multiple functions have been discovered to also influence appetitive behaviors and energy balance. Examples include bile acids [52], glucagon [53], and amylin [54], all shown to influence food intake through central signaling mechanisms. To add to the complexity, these hormones, intestinally derived factors, insulin and leptin, often exhibit ultradian and diurnal patterns of appearance, disruption of which can augment or diminish their signals [55]. Finally, all these signals and systems operate within a circadian framework that, when altered, can affect normal food intake and body weight [56]. One way to make sense of the impact of these physiological mechanisms is to consider the “state” as either being pre‐ or postprandial and the “trait” as being above, at, or below the adipose mass set point.

#### Appetitive behaviors follow changes in gut signals in adaptation to low‐calorie dieting

As noted above, studies of patients undergoing low‐calorie diet‐induced weight loss will have adaptive downregulation of satiety hormone levels and central signaling, and enhanced secretion of the orexigenic hormone ghrelin. These changes and those in other intestinally derived gut signals, as well as reductions in leptin levels in the context of leptin resistance, lead to increased patient‐reported hunger levels and lower fullness scores (Fig. 2) [48]. Patients commonly report this as “food noise” [57]. In this context, it is important when counseling patients on expectations to distinguish which behaviors are physiologically driven and those that are more culturally or consciously controlled, such as food choices (e.g., ultra‐processed snacks and foods vs. whole foods and healthier snacks). The former will most likely be amenable to pharmacologic and surgical treatments, whereas the latter will be important targets of behavioral approaches that complement drug effectiveness.

### Implications for treatment

These homeostatic adaptation responses to low‐calorie‐induced weight loss are complex and potent. They remain in place until adipose mass is regained and the adipose mass set point is restored, even when it resides at a level above what is considered a healthy range. These responses are the reasons that calorie restriction alone is ineffective as a long‐term approach to treat the disease of obesity [10]. Patients who regain adipose mass in this context do so not because they have become noncompliant, but because their biology counters their efforts. Unless properly counseled and volitional calorie restriction is avoided, this approach risks reinforcing patient feelings of inadequacy and failure as well as external and internal obesity stigma. Patients who reduce adipose mass through dieting and then gain it back again are not being recalcitrant; they are simply inadequately treated by their provider.

#### Nutritional (lifestyle) therapies compared with calorie restriction

Moving beyond low‐calorie dieting to include a behavioral lifestyle intervention, which consists of counseling, regular dietician visits, and increased activity, results in a better long‐term weight loss response but one that is still very modest. Several such programs have been reported, with one of the most widely cited being the Diabetes Prevention Program [58]. In this study, patients with a BMI above 24 kg/m^2^ and prediabetes were randomized to a weight loss lifestyle intervention versus placebo or metformin. At the end of 1 year, the lifestyle intervention arm lost an average of 7% total body weight [58]. This backtracked to 4% total weight loss after 4 years and then 2% after 10 years of follow‐up, at which time it was no longer different from placebo [59]. Despite these averages, there were a smaller number of patients who achieved and maintained more substantial weight loss [60]. These patients most likely represent a biological “responder” subset to lifestyle, similar to “salt‐sensitive” hypertensives, and thus the approach in their case was most likely an effective treatment for their specific disease type of obesity.

Documented within this trial was compliance with treatment group assignment for the first 4 years of study, so the backtracking in weight in the lifestyle group can be ascribed to adaptation responses. However, this very small long‐term weight loss from lifestyle alone does not negate the benefits of these nutritional and exercise therapies on the health of patients who adopt and maintain them. Indeed, continuing the lifestyle treatments, even if only for the 4% weight loss at 4 years, was still associated with up to a 58% reduction in risk of developing type 2 diabetes [61]. Instead, nutritional and exercise therapies are considered important in the management of patients with obesity, but for most patients, they will not be sufficient by themselves to achieve more meaningful weight loss and additional health benefits. In the smaller number of patients for whom their disease of obesity adequately responds, these treatments may be sufficient. For the remaining, majority “nonresponders,” obesity medications and surgeries should no longer be considered “adjuncts” (i.e., optional) to lifestyle interventions but necessary next steps in the management of their disease.

#### Obesity medications

With the exception of orlistat, current obesity medications are all understood to work through the subcortical areas of the brain, including the brainstem, to alter the adipose mass set point [62]. When treatment is started and effective, the patient's brain perceives them to be above the adipose mass set point, and hence, the subcortex of the brain will use the biological levels available to bring adipose mass into homeostasis. Initially, an improvement in appetitive behaviors conducive to sustained reduction in calorie intake often precedes weight loss [63, 64]. By countering the adaptive response by the brain in response to the changes in gut hormone levels and reduction in leptin levels, obesity medications allow for homeostatic adipose mass regulation at a lower calorie intake and adipose mass set point [65, 66, 67]. This is not a permanent resetting, as medication discontinuation has consistently shown return of hunger and adipose mass regain [68, 69]. Hence, obesity medications are recommended to be continued long‐term.

**Fig. 3:**
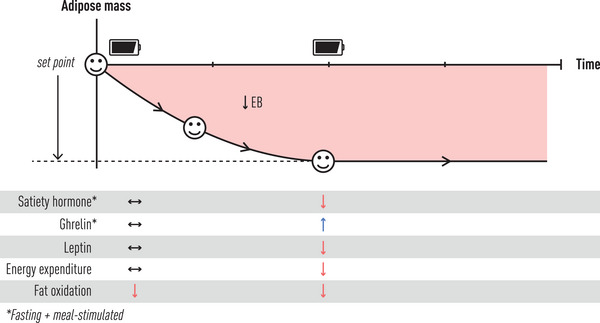
Metabolic, hormonal, and appetitive adaptive responses to obesity‐medicine‐induced weight loss. Facial expressions indicate patient experiences from baseline adipose mass set point to during active negative energy balance (EB), during restoration of a new, lower set point. See text for full descriptions.

Preclinical data support obesity pharmacology's role to favorably adjust adipose mass homeostasis (Fig. 3). Understanding the changes in bodyweight, food intake, and energy expenditure in rodents treated with medications, such as the combination of cagrilintide and semaglutide [70] or amycretin [71], will help clinicians educate patients about what to expect and why they will experience changes in appetitive behavior. The amount of time that rodents need to move from one state of homeostasis (pre‐intervention) to a new state of homeostasis (post‐intervention) appears to be between 20 and 30 days, whereas the same process takes up to 12–18 months in humans [72, 73]. The patterns of biological changes, however, appear to be similar. In rodents, immediately after treatment initiation, food intake substantially reduces to 30%–40% of baseline for 1–6 days. This coincides with the most rapid weight loss phase. After 6 days, food intake increases substantially to 70%–80% of baseline, although bodyweight continues to decline. After 20 days, food intake returns close to but below baseline (80%–90% of baseline), albeit at a bodyweight that now remains constant in a new homeostatic set point. This often occurs roughly 20% below baseline bodyweight (alternatively calculated 80% of baseline weight) and indicates maximal drug effectiveness. During the same 20–30‐day period, energy expenditure of the rodents only reduces slightly from baseline and remains constant at around 80%–90% of baseline as long as medications are continued [70]. It appears energy expenditure goes to where it should be to achieve homeostasis, whereas food intake is more dynamic, with excessive reductions for the first 6 days and then a gradual return to where it should be to maintain homeostasis between days 6 and 20. An oversimplified representation would be that with these medications, homeostasis is reached when 80% of baseline weight is achieved with 80% of food intake at baseline, and 80% of energy expenditure at baseline. In rodents, the gene activation in the liver and muscle during this process is subtle, suggesting the rodents are reducing their adipose mass homeostasis without physiological counter regulation because the biological processes are aligned to achieve homeostasis. This contrasts significantly with rodents that are calorie restricted to achieve the same body weight loss where food intake must be much lower because energy expenditure is greatly reduced and biological counter regulation is evident by gene activation in the liver and muscle [70]. Similar preclinical data have been generated for tirzepatide [67], but findings in humans undergoing short‐term calorie reduction treated with drug versus placebo demonstrated both similarities and differences. Like the rodents, tirzepatide treatment in humans markedly reset (lowered) appetitive behavior to be appropriate for the new, lower body weight [67]. However, unlike the rodents, energy expenditure adaptation to weight loss was not improved, but rather drug treatment increased fat oxidation compared to placebo during the weight reduction phase [67]. This shows that studies in humans when they achieve homeostasis in the weight maintenance phase are still needed to understand how current and future drug modalities will impact energetic, hormonal, and appetitive adaptation responses to adipose mass loss. Therapies that favorably impact more than one of these pathways likely will have the best chance for meaningful and durable weight loss success.

This framework creates an ability to understand the preclinical and clinical data in which adipose mass regulation is biologically determined and can be changed by effective obesity treatments. This also explains the transition from substantial reduction in appetitive behavior during the dynamic weight reduction phase to the perceived return of appetitive behavior during the weight maintenance phase. Rather than being a source of disappointment or concern, this can be conveyed as the expected response to reaching maximal drug effectiveness.

#### Metabolic–bariatric surgery

Metabolic–bariatric surgery remains a highly effective approach to achieve meaningful control of the disease of obesity, including amount, duration, and associated health improvements of weight loss, and sustained weight loss. For the most recommended procedures (sleeve gastrectomy, Roux‐en‐Y gastric bypass, and biliopancreatic diversion), multiple changes in gut‐derived factors governing appetitive behaviors have been identified that favor not only initial reductions in food intake [74] but also prevent the expected adaptive hormonal, appetitive behaviors, and metabolic (energetic) changes necessary for long‐term weight loss maintenance [75, 76]. These findings demonstrate that metabolic–bariatric surgeries are effective when they successfully alter endogenous signals that recapitulate the biological processes described above to pharmacologic treatments of obesity, with the new adipose mass homeostasis taking 12–18 months to be achieved. During this initial weight reduction phase, food intake is reduced [75], whereas energy expenditure reduction remains less than would be expected from calorie restriction [76]. When adipose mass homeostasis is again achieved, food intake increases, and food preferences return toward baseline, but body weight remains stable at the newly reduced homeostatic point [75]. In 8%–20% of patients who undergo metabolic–bariatric surgery, the disease of obesity progresses (or response to treatment is lost) and weight regain may occur after one to two years; however, in the majority of patients, the adipose mass homeostasis is maintained at 35%–40% lower levels [77].

#### Impact of reduction in adipose mass on skeletal muscle mass

As body weight is a composite of adipose, organ, and skeletal muscle mass, the impact of weight loss on skeletal muscle mass reduction should also be considered. The homeostatic amount of muscle required to support a patient carrying excess adipose mass at a weight of 100 kg is substantially more than the muscle mass required to support a patient with reduced adipose mass weighing 75 kg. Thus, a reduction in skeletal mass may be expected as part of the newly reduced adipose mass homeostasis regardless of treatment approach. Studies of patients treated with obesity medication and metabolic–bariatric surgery skeletal muscle have shown muscle mass reductions [78], but physical functioning improves [79]. The concern regarding excessive skeletal muscle mass reduction even in vulnerable populations, such as the elderly, has not yet been substantiated, but if the biological processes are better understood, then it may be possible to intervene with nutritional (higher protein) [80], exercise (resistance), or pharmacological treatments (anti‐myostatin [81]) to protect muscle mass. This may result in further functional improvement.

### Summary and identification of key areas in need of future research

Expression and maintenance of the disease of obesity is rooted in a complex biology, including appetitive and energetic adaptations to weight loss as part of the homeostatic regulation of adipose mass. Understanding this biology has led to the development of highly effective obesity medications and weight‐loss inducing surgical procedures that allow a patient to transition from one homeostatic adipose mass set point to another, ranging from up to 20% (with newer drugs) to 30% (following metabolic–bariatric surgery) lower in body weight based on current evidence.

When effective, both animal models and human studies indicate these treatments initially alter endogenous hormonal signals to reduce appetitive behaviors. This leads to improvements in feelings of hunger and fullness, resulting in ingestive behaviors conducive to smaller portion sizes and weight loss. The successes of newer obesity medications and metabolic–bariatric surgeries derive from their ability to interfere with the enhanced biological signals promoting food intake normally elicited as part of the homeostatic adaptation response to weight loss, including alterations in meal‐related gut hormone release and leptin levels. Patients often report this as a reduction in “food noise,” and it is associated with an improvement in their quality‐of‐life measures [57].

At the most fundamental level, both obesity medicines and metabolic and bariatric surgery are effective because they treat the disease of obesity by enhancing leptin signaling or leptin sensitivity, manifested when a new homeostatic set point is sustained at a lower, healthier adipose mass. Identification of other adiposity and body composition signals is important to better understand biological responses to adipose mass changes and their impact on expression of adiposity‐associated complications. Key among these signals will be identifying those that enhance leptin signaling (leptin “sensitizers” [82]) and the governance of skeletal muscle mass, the pharmacologic regulation of which has already demonstrated favorable maintenance of lean mass during weight loss and enhanced adipose mass loss [83], effectively serving as weight‐loss “signal multipliers.” Ultimately, understanding obesity as a disease and how homeostasis of adipose mass can be altered and maintained will result not just in improved patient health, but also a better patient experience and quality of life.

## Conflict of interest statement

ClR reports grants from the EU Innovative Medicine Initiative, Irish Research Council, Science Foundation Ireland, Anabio, and the Health Research Board. He serves on advisory boards and speakers panels of Novo Nordisk, Roche, Herbalife, GI Dynamics, Eli Lilly, Johnson & Johnson, Gila, Irish Life Health, Boehringer Ingelheim, Currax, Zealand Pharma, Keyron, AstraZeneca, Arrowhead Pharma, Amgen, AbbVie, Metsera, Nymble, Olympus, and Rhythm Pharma. ClR is the Chair of the Irish Society for Nutrition and Metabolism. ClR received stock options as payment for scientific advisory board functions from Metsera and Nymble. ClR provides obesity clinical care in the My Best Weight clinic and Beyond BMI clinic and is a co‐owner of these clinics. J.Q.P. has received consulting fees from Boehringer Ingelheim and Novo Nordisk.
